# Changing Risk Factors in Patients Diagnosed with Human Immunodeficiency Virus

**DOI:** 10.5811/westjem.52947

**Published:** 2026-08-26

**Authors:** Sula Frausto, Heather Sperring, Glorimar Ruiz-Mercado, Cassandra Pierre, Kathryn Scrudder, Ijeoma Okafor, Kerrie Nelson, Elissa M Schechter-Perkins

**Affiliations:** * Boston Medical Center, Department of Emergency Medicine, Boston, Massachusetts; † Boston Medical Center, Section of Infectious Diseases, Boston, Massachusetts; ‡ Boston University Chobanian & Avedisian School of Medicine, Boston, Massachusetts; § Boston University School of Public Health, Department of Biostatistics, Boston, Massachusetts

## Abstract

**Introduction:**

Since 2019, transmission risk factors among patients diagnosed with HIV have changed, although the trends and reasons behind them have not been fully elucidated. The objective of this study was to assess changes in HIV transmission risk factors among patients newly diagnosed with HIV to inform future targeted HIV testing programs.

**Methods:**

This study site is New England’s largest essential hospital and a major reporter of new HIV cases in Massachusetts. We performed a retrospective chart review of patients diagnosed with HIV infection at our medical center between January 2019 and December 2023. Demographic and clinical variables were collected and analyzed using descriptive and comparative statistics to evaluate trends in primary risk factor for HIV. Our primary outcome measure was incident HIV diagnosis with a primary risk factor of injection drug use (IDU), men who have sex with men (MSM), or heterosexual contact. A secondary outcome examined was CD4 count at the time of diagnosis.

**Results:**

A total of 246 patients were newly diagnosed with HIV. Of these, 59% (*n* = 146) reported heterosexual contact as their most likely cause of transmission, 19% (*n* = 47) MSM, 19% IDU (*n* = 46), and 3% (*n* = 7) reported both MSM and IDU. Heterosexual contact increased as the predominant HIV transmission factor from 53% (24/45) in 2019 to 79% (37/47) in 2023 (risk difference, 25%; 95% CI, 6.7%–44.1%), and the percentage of diagnoses made in non-US-born patients increased from 60% (27/45) in 2019 to 83% (39/47) in 2023 (risk difference, 23%; 95% CI, 5.1%–41%). Non-US born patients had significantly lower CD4 counts at diagnosis (mean [SD] CD4 count 299 [241] cells/μL) compared to US-born counterparts (mean [SD] CD4 count 498 [309] cells/μL, *P* < .001), with a mean difference of −199 cells/μL (95% CI, −272 to −125).

**Conclusion:**

The dominant risk factor for HIV transmission has changed from injection drug use/men who have sex with men to heterosexual contact in New England’s largest city. Our findings associate increased HIV incidence in non-US-born immigrants at an urban essential hospital, highlighting the importance of considering this group in targeted public health efforts to avoid missing cases of HIV in similar settings.

## INTRODUCTION

Since the onset of the HIV epidemic in the early 1980s, an estimated 91.4 million people have been infected with the virus.^1^ Although the incidence of HIV in the United States had decreased by nearly 76% from 1984 to 2022, 2,3 a comprehensive understanding of the factors underlying current HIV transmission remain unknown. The US Centers for Disease Control and Prevention (CDC) HIV Surveillance Reports stratify transmission rates nationally based on factors such as age, gender, race/ethnicity, and transmission category (including men who have sex with men [MSM] and injection drug use [IDU]); however, they do not report on HIV incidence by an individual’s country of origin.^3^ While these data are often difficult to obtain, it is crucial to gain a broader understanding of how HIV transmission in the United States may be associated with migration from various countries or regions of origin. It is already known that HIV transmission risk factors in non-US-born individuals differ in proportion compared to US-born counterparts. Non-US-born individuals have higher rates of reporting heterosexual contact as their primary transmission risk factor, with lower rates of MSM and IDU.^4^,^5^ We do not know, however, whether there are temporal associations in trends between overall primary HIV transmission risk factor, foreign-born status, and disease severity within the US.

In February 2019, the US Department of Health and Human Services (HHS) published their plan, “Ending the HIV Epidemic: A Plan for America,” to end the HIV epidemic by the year 2030.^6^ In that plan, Suffolk County, MA, was identified as one of 48 “high burden counties” in which HHS efforts would be prioritized. Our institution, located within Suffolk County, accounts for 27% of all new HIV diagnoses in Massachusetts.^7^ The objective of the current study was to assess risk factors for new HIV diagnoses among patients at an urban essential hospital in Boston, MA, as well as to elucidate trends and associations between risk factors and demographic variables including country of origin, disease severity, and comorbid diagnoses.

## METHODS

### Setting

Our institution is a 514-bed tertiary academic medical center in Suffolk County, MA. It is New England’s largest essential hospital and diagnoses more new HIV cases per year than any other institution in the state (personal communication; Johns B. Director, Division of STD Prevention and HIV Surveillance, Massachusetts Department of Public Health [MDPH], October 2, 2020). As such, this study site is uniquely poised to identify regional trends in HIV risk factors.

### Study Design and Data Collection

This study was deemed exempt by the institutional review board (IRB). It is a retrospective chart review that adheres to previously published methodological guidelines for retrospective medical record reviews.^8^ Specific methods used include abstractor training, case selection criteria, variable definitions, performance monitored, medical record identified, sampling method, missing-data management plan, and IRB approval. It is a retrospective review of all patients diagnosed with incident HIV infection at our institution between January 1, 2019, and December 8, 2023. Tests are done at the request of the patient or based on physician discretion, and verbal consent is required prior to testing. Patients with a confirmed HIV diagnosis were identified via a report generated by our institution’s clinical laboratory, and manual chart abstraction from both the electronic health record and the Integrated Testing and Linkage Report (created at time of HIV appointment intake) was performed for patient demographic and clinical information. We excluded from the study individuals younger than 18 years or newly diagnosed with HIV.

**Population Health Research Capsule**

What do we already know about this issue?

*HIV transmission patterns have changed over time, but evolving trends in recent years remain insufficiently described*.

What was the research question?

*How have transmission risk factors and demographics changed for incident HIV diagnoses from 2019 to 2023?*

What was the major finding of the study?

*Heterosexual transmission rose 53% to 79% (RD 25%; 95% CI, 6.7–44%; P < .05); non-US-born cases rose 60% to 83% (RD 23%; 95% CI, 5.1–41%; P < .05)*.

How does this improve population health?

*Findings identify rising risk in non-US-born groups to guide targeted, culturally appropriate HIV testing and earlier diagnosis*.

Demographic variables collected include age, sex, race, ethnicity, country of origin, and housing status; all variables except age were self-reported. We categorized non-US-born individuals into one of five global regions of their origin (Africa, Caribbean, Asia, Latin America, Europe) and included those born in US dependencies as non-US-born. Clinical variables of interest include the primary cause of HIV infection (IDU, MSM, heterosexual intercourse), CD4 count at the time of diagnosis (cells/μL), if available, and presence/absence at time of diagnosis of the following: syphilis; hepatitis C virus; tuberculosis; pregnancy; and psychiatric diagnoses. Patients with no CD4 count recorded at the time of diagnosis were excluded from summary statistics. Reporting of risk factors for HIV infection is required by the state for offering government-funded HIV services. For individuals with more than one risk factor for HIV infection, the most likely primary cause of HIV infection was adjudicated by data collectors according to the MDPH algorithm, which follows the CDC hierarchical ranking method.^9^ We used REDCap electronic data capture tools (hosted at CTSI 1UL1TR001430) and Excel for data collection and management.^10^

### Patient and Public Involvement

Patients or members of the public were not involved in the design, conduct, reporting, or dissemination of our research.

### Data Analysis

We used descriptive statistics to report the demographic and clinical characteristics of patients newly diagnosed with HIV at an urban essential hospital between January 2019 and December 2023. The Cochran-Armitage trend test was used to determine the trend of new patient HIV diagnoses per year, an ordinal variable, over a five-year time frame for each of the dichotomous demographic and clinical characteristics. Due to the small sample sizes, it was decided to conduct the statistical tests on the yearly HIV patient numbers rather than the monthly numbers. We performed chi-square tests (categorical) and two independent sample *t*-tests (continuous) to evaluate associations between the likely primary risk factor for infection and clinical characteristics of patients. Alternative nonparametric tests, including the Fisher exact test (categorical), were used when assumptions for using parametric tests were not met. Additionally, we used analysis of variance when the demographic variable (continuous) was being compared between more than two groups. To correct for multiple testing issues, the *P* values obtained from hypothesis tests were Bonferroni adjusted by the number of statistical tests performed. We analyzed data with SAS software version 9.4 (SAS Institute Inc, Cary, NC).

## RESULTS

Between January 1, 2019, and December 8, 2023, a total of 103,759 HIV tests were administered across our institution. Of the 103,759 tests performed, 246 patients (0.24%) were identified as newly diagnosed with HIV and included in this study. Of the 7,371 tests performed in the emergency department (ED), 63 (0.85%) were identified as newly diagnosed with HIV. The frequency of new HIV diagnoses made at our institution in yearly intervals is summarized in Figure 1. Demographic and clinical characteristics of those newly diagnosed with HIV by year of diagnosis are summarized in Table 1.

The median age of the 246 patients included was 37 years (interquartile range 30–39), with 62.2% identifying as male, 63% identifying as Black, 17.9% identifying as White, 17.9% identifying as Hispanic or Latinx, and 17.9% identifying as “other” race or declining to identify their race. Other than the United States (*n* = 94), the most represented countries of origin include Haiti (*n* = 62), the Dominican Republic (*n* = 12), and Cape Verde (*n* = 9). Patients born in Haiti made up 40% of non-US-born diagnoses and 25% of all new HIV diagnoses at our institution within the study period. Of all included patients, 42% (*n* = 103), were non-English speakers, in contrast to our institution’s overall patient population, of whom 30% speak a primary language other than English.

From the Cochran-Armitage trend tests of the study population demographics in Table 1, we observed statistically significant trends in the proportions of language (*P* = .002), country region (*P* = .01), risk factors (*P* = .006), and psychiatric comorbidities (*P* = .009) across the study years (2019–2023). We observed increasing linear trends in HIV diagnoses among unstably housed patients, non-English speakers, patients from the Caribbean, and those likely infected through heterosexual contact during the five-year study period (all *P* values < .05). Conversely, we observed decreasing linear trends in HIV diagnoses among White patients and patients from the US, as well as those with psychiatric comorbidities, hepatitis C, and patients whose HIV transmission was likely from MSM, IDU, and MSM/IDU (all *P* values < .05). No statistically significant linear trends were observed over the study period for age, gender, ethnicity, diagnosing department, CD4 count, or presence or absence of syphilis, tuberculosis, and pregnancy at the time of diagnosis.

Table 2 presents the clinical characteristics of the total patient sample and by the most likely cause of their HIV infection. Statistically significant associations were found between primary HIV risk factor, CD4 counts (*P* < .001), psychiatric comorbidities (*P* < .001), hepatitis C (*P* < .001), and syphilis (*P* < .001). Supplemental Table 2 indicates that patients infected with HIV primarily due to heterosexual contact or MSM have a significantly lower CD4 count at the time of diagnosis compared to those reporting IDU (mean difference = −318 cells/μL; 95% CI, −483 to −197; and mean difference = −227 cells/μL; 95% CI, −376 to −79), respectively).

With respect to other demographic characteristics, the mean CD4 count at the time of diagnosis was significantly higher among non-US-born patients compared to US-born patients (mean difference, 199 cells/μL; 95% CI, 125–272). Similarly, patients diagnosed outside the ED had significantly higher CD4 counts at diagnosis than those diagnosed in the ED (mean difference, 145 cells/μL; 95% CI, 59–230), as shown in Table 3.

## DISCUSSION

This study is a retrospective chart review of 246 patients with incident HIV infection from January 2019 to December 2023. Ultimately, we found that over the five-year study period, heterosexual contact increased in prominence as the predominant HIV transmission risk factor. Consequently, we noted that IDU as a primary transmission risk factor has been decreasing since 2020, as have hepatitis C codiagnoses, psychiatric comorbidities, and unstable housing status. These variables may be linked, as patients seen at our institution who inject drugs are often unstably housed, have co-occurring alcohol, substance, or opioid use disorders, and have existing hepatitis C diagnoses from IDU. It is also worth noting that CD4 count at the time of diagnosis was significantly associated with both primary transmission risk factor and housing status, with IDU and those who are unstably housed showing higher CD4 counts than average. These associations suggest that patients with IDU or unstable housing may be diagnosed with HIV closer to the time of infection than other high-risk groups, which may be due to greater use of the ED among patients with these risk factors; however, further studies are needed to understand the reasons for these associations.

It is striking that although only 7.1% of HIV tests at our institution were performed in the ED, 25.6% of the diagnoses of the virus were made in the ED, and the CD4 counts of individuals diagnosed in the ED were significantly lower than those diagnosed elsewhere in the institution. A high prevalence of HIV in ED patients is consistent with prior literature, and a major reason why multiple guidelines across the US have, since 2006, recommend ED HIV screening as a major component of ending the HIV epidemic.^11^–^13^

As seen in Figure 3, non-US-born individuals now make up 75% and 83% of new HIV infections diagnosed in 2022 and 2023, respectively, which is a statistically significant trend by year. We found that there has also been a statistically significant association between language and year, with non-English speakers now making up most new HIV cases, which may be associated with the increase in diagnoses among non-US-born patients. While foreign-born status is not a transmission risk factor for HIV, it is an illuminating piece of context that can inform future HIV prevention efforts.

When comparing our results to trends reported by the MDPH Bureau of Infectious Disease and Laboratory Sciences from 2019 to 2021, we find that our data align by foreign-born status, but not gender, race, or risk factor.^14^ The MDPH reports a lower incidence among women, Black non-Hispanic individuals, and people reporting heterosexual transmission than we found at our institution. This trend may be in part due to the fact that our institution is considered an essential hospital—meaning we provide care to a high volume of uninsured and vulnerable patients, regardless of their ability to pay or immigration status. Previous studies have demonstrated that heterosexual contact as an HIV transmission risk factor is higher in non-US-born individuals than their US-born counterparts, as well as higher transmission among women, which may account for the trends present at our institution.^4^,^5^

The CDC’s National HIV Surveillance System (NHSS) reports demographic characteristics and transmission risk category from data collected by local health jurisdictions.^3^ When examining the most recent of these reports, which includes HIV incidence among individuals 13 years of age and above from 2018 to 2022, we found both similarities and differences compared to our results. The NHSS reports a significant 12% decrease in male diagnoses from 2018 to 2022, compared to our finding, which was not statistically significant; and that male diagnoses decreased by 27% from 2019 to 2023 (*P* =.107). The NHSS did not find a significant increase in female diagnoses, and used sex assigned at birth rather than current gender identity. The NHSS also found a significant 10% decrease in HIV incidence for those reporting MSM as their only transmission risk factor between 2018 and 2022, with no change detected for those reporting IDU or heterosexual contact. The NHSS does not report HIV incidence by US- versus non-US-born status. These similarities and differences may be due to our limited sample size or the patient population specific to our institution. However, it is possible that while the trends demonstrated here regarding transmission among non-US-born individuals may be a national trend, they cannot be compared to nationally reported data because these variables are not collected.

It is important to note that this study is particularly relevant to other urban HIV epicenters where there has been a recent immigration boom, including Denver, CO, Chicago, IL, and New York City, NY. These states had a 540% increase, 540% increase, and 1020% increase, respectively, in US Customs and Border Protection encounters from 2021 to 2023,.^15^ Thus, further research investigating HIV transmission risk factors should be conducted in other priority jurisdictions outlined by the Ending HIV Epidemic Initiative, and leaders of public health interventions should consider focusing their efforts on these areas.

The increase in HIV incidence among non-US born individuals has occurred against the backdrop of limited opportunities for HIV detection, treatment and prevention upon arrival in the US. Prior to 2010, HIV testing was mandatory for immigrants at the time of entry to the US as part of their immigration examination. Criticisms of the ban soon mounted, with opponents noting that HIV is not transmissible via casual contact and that at the time, the US had more known cases of HIV than any other country. The removal of the ban in effect in early 2010 had far-reaching positive effects ranging from decreasing the stigma faced by those with HIV to allowing for their safer travel, since they no longer had to fear being deported if medications were found during the immigration process. However, lifting mandatory HIV entrance testing removed a critical touchpoint for new migrants to receive testing and linkage to care.^16^

While it is difficult to quantify the impact of no longer mandating HIV entrance testing, HIV incidence in the US decreased by 10.3% in the four years following the policy change, which may reflect lower rates of detection among immigrants arriving to the US.^17^ Furthermore, recently arrived immigrants may face ongoing socioeconomic barriers to healthcare access that may preclude earlier entry to HIV care along the route of migration as well as in the US.^18^ When examining existing barriers to testing among immigrants, studies suggest that many immigrants express fear that testing positive for HIV will result in deportation, with some citing the former HIV travel ban as a key contributor to their hesitancy to test.^19^

Non-US-born patients had significantly lower CD4 counts at the time of diagnosis compared to their US-born counterparts. This trend may be associated with a delay between HIV infection and testing for non-US-born patients, potentially due to the lengthy establishment of care upon arrival to the US. However, several studies suggest that many non-US-born individuals acquire HIV after immigrating to the US.^20^,^21^ As suggested by Harawa et al, it is difficult to accurately characterize the time at which immigrants are infected, as many travel back and forth between their country of origin and the US. Among foreign-born Black men, lack of familiarity with the US healthcare system, low risk perception, and a lack of testing services advertised in their native language may contribute to their decreased likelihood of receiving HIV screening.^22^

## LIMITATIONS

This study was performed at a single-site urban essential hospital and, therefore, results may not be generalizable to other institutions or geographic areas. Additionally, data collection was done through a retrospective chart review. Not all variables, particularly co-occurring diagnoses and housing status, were available for each patient, especially those who had recently immigrated and had not established care in the US prior to HIV diagnosis. Many of these patients were also not tested for the co-occurring diagnoses of interest at the time of HIV screening. Unfortunately, we lack data regarding where non-ED diagnoses were made in our institution, which may affect the proportions of transmission risk factors and other variables presented here. Due to our small sample size, the presence of psychiatric comorbidities was documented as a broad category rather than individual diagnoses, sinces we would be less likely to draw any associations. Furthermore, we do not know whether the psychiatric comorbidities documented in each patient’s chart were active diagnoses at the time of HIV testing and diagnosis.

With the exception of MSM co-occurring with IDU, only one primary risk factor for HIV infection was documented per patient and was determined according to an algorithm outlined by the MDPH for HIV data collection. However, transmission risk factors are not mutually exclusive. Furthermore, risk factors are self-reported and, thus, may also be affected by one’s country of origin, as lingering stigmas against MSM and IDU from their home country and the U.S. may influence their willingness to disclose either as their primary risk factor. Moreover, data were not consistently available about the country of origin of our institution’s overall patient population, making conclusions about the proportionality of HIV infection by country of origin difficult to ascertain. Finally, there is no universal opt-out HIV screening program at our institution. Patients are tested at their request or based on physician discretion, which requires verbal consent prior to testing; thus, there may be patients at this institution who had incident HIV during the study period and were not included in the current analyses.

## CONCLUSION

Our study may suggest that incident HIV diagnoses among non-US-born immigrants reporting heterosexual contact have become a primary HIV transmission demographic, which has not been fully explored before. Our findings suggest that targeted public health efforts be directed at non-US-born immigrants, particularly those from Latin America and the Caribbean. Increased testing among these groups is a must; however, we must be careful not to stigmatize them further. Therefore, culturally and linguistically relevant immigration education, testing, and linkage-to-care outreach efforts should be prioritized.

One final point of advocacy is for universal opt-out HIV testing. The CDC currently recommends routine opt-out HIV screening, as it mitigates stigma associated with testing, facilitates earlier diagnosis in overlooked groups, and ultimately decreases chances of transmission.^23^ However, each state has its own laws governing HIV testing; in Massachusetts, separate verbal informed consent is required prior to initiating HIV testing. Language barriers and stigma may, therefore, have a greater impact on testing acceptance in Massachusetts than in other states.^24^ Should universal HIV opt-out testing become available across healthcare settings, stigma about HIV testing and transmission of HIV among the high-risk populations outlined in this paper may be greatly reduced. Future research should be aimed at investigating these emerging trends in HIV risk factors across multiple study sites and geographic locations.

## Supplementary Information





## Figures and Tables

**Figure 1:**
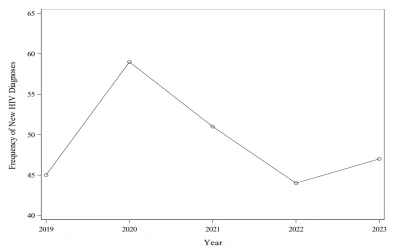
Time series plot showing frequency of new HIV diagnoses between January 1, 2019–December 8, 2023 at an urban essential hospital, by year, in a study assessing changes in transmission risk factors among patients newly diagnosed with the virus.

**Figure 2:**
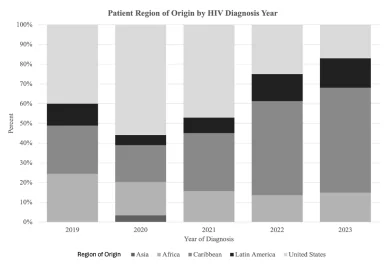
Region of origin of patients diagnosed with HIV by diagnosing year in a study on the changing trends in risk factors among patients diagnosed with the virus. Those born in US dependencies are not categorized as US-born, but rather as their geographic location. There was a 21% increase in non-US-born patients newly diagnosed with HIV between 2019 and 2022 (*P* = .01).

**Table 1:** Longitudinal trends in demographic and clinical characteristics of newly diagnosed HIV patients at an urban essential emergency department overall and by year of diagnosis 2019–2023 (*N* = 246).

Characteristics	Total Sample *N* = 246	Diagnosed 2019 *n* = 45	Diagnosed 2020 *n* = 59	Diagnosed 2021 *n* = 51	Diagnosed 2022 *n* = 44	Diagnosed 2023 *n* **=** 47	*P* value
Age							
Mean (SD)	40.02 (12.72)	39.07 (12.77)	38.51 (12.15)	41.65 (13.91)	42 (13.19)	39.19 (11.63)	>.999
Median (IQR)	37 (30 – 49)	37 (30 – 49)	35 (29 – 47)	40 (30 – 51)	38.5 (32.5 – 50.5)	38 (30 – 45)	
Min, Max	18, 75	21, 68	21, 69	18, 74	21, 75	20, 69	
Gender, *n* (%)							
Male	153 (62.20)	34 (75.56)	37 (62.71)	32 (62.75)	27 (61.36)	23 (48.94)	>.999
Female	90 (36.58)	11 (24.44)	22 (37.29)	17 (33.33)	16 (36.36)	24 (51.06)	
Transgender male	1 (0.41)	0 (0.00)	0 (0.00)	1 (1.96)	0 (0.00)	0 (0.00)	
Transgender female	2 (0.81)	0 (0.00)	0 (0.00)	1 (1.96)	1 (2.27)	0 (0.00)	
Race, *n* (%)							
White	44 (17.89)	7 (15.56)	20 (33.9)	8 (15.69)	6 (13.64)	3 (6.38)	.197
Black	155 (63.01)	30 (66.67)	26 (44.07)	37 (72.55)	29 (65.91)	33 (70.21)	
Asian	2 (0.81)	0 (0.00)	2 (3.39)	0 (0.00)	0 (0.00)	0 (0.00)	
Native Hawaiian or Other Pacific Islander	1 (0.41)	1 (2.22)	0 (0.00)	0 (0.00)	0 (0.00)	0 (0.00)	
Other	44 (17.89)	7 (15.56)	11 (18.64)	6 (11.76)	9 (20.45)	11 (23.40)	
Ethnicity, *n* (%)							
Not Hispanic or Latinx	202 (82.11)	37 (82.22)	51 (86.44)	44 (86.27)	34 (77.27)	36 (76.6)	>.999
Hispanic or Latinx	44 (17.89)	8 (17.78)	8 (13.56)	7 (13.73)	10 (22.73)	11 (23.4)	
Language, *n* (%)							
English speaker	143 (58.13)	30 (66.67)	46 (77.97)	33 (64.71)	19 (43.18)	15 (31.91)	.002
Non-English speaker	103 (41.87)	15 (33.33)	13 (22.03)	18 (35.29)	25 (56.82)	32 (68.09)	
Housing Status, *n* (%)							
Stable	171 (69.51)	35 (77.78)	29 (49.15)	41 (80.39)	30 (68.18)	36 (76.6)	.062
Not stable	63 (25.61)	8 (17.78)	27 (45.76)	6 (11.76)	12 (27.27)	10 (21.28)	
Unknown	12 (4.88)	2 (4.44)	3 (5.08)	4 (7.84)	2 (4.55)	1 (2.13)	
Country Region, *n* (%)							
Asia	2 (0.81)	0 (0.00)	2 (3.39)	0 (0.00)	0 (0.00)	0 (0.00)	.012
Africa	42 (17.07)	11 (24.44)	10 (16.95)	8 (15.69)	6 (13.64)	7 (14.89)	
Caribbean	83 (33.74)	11 (24.44)	11 (18.64)	15 (29.41)	21 (47.73)	25 (53.19)	
Latin America	25 (10.16)	5 (11.11)	3 (5.08)	4 (7.84)	6 (13.64)	7 (14.89)	
United States	94 (38.21)	18 (40.00)	33 (55.93)	24 (47.06)	11 (25.00)	8 (17.02)	
Diagnosing Department, *n* (%)							
ED	63 (25.61)	13 (28.89)	14 (23.73)	10 (19.61)	10 (22.73)	16 (34.04)	>.999
Non-ED	183 (74.39)	32 (71.11)	45 (76.27)	41 (80.39)	34 (77.27)	31 (65.96)	
**Clinical Characteristics**							
Most likely cause of HIV transmission, *n* (%)							
Heterosexual contact	146 (59.35)	24 (53.33)	26 (44.07)	29 (56.86)	30 (68.18)	37 (78.72)	.006
MSM	47 (19.11)	14 (31.11)	8 (13.56)	13 (25.49)	6 (13.64)	6 (12.77)	
IDU	46 (18.70)	4 (8.89)	22 (37.29)	9 (17.65)	7 (15.91)	4 (8.51)	
MSM/IDU	7 (2.84)	3 (6.67)	3 (5.08)	0 (0.0)	1 (2.27)	0 (0.0)	
† CD4 count at time of diagnosis (cells/μL)							
Patients with available CD4 count (*n*)	222 (90.24)	39 (86.67)	54 (91.53)	46 (90.20)	38 (86.36)	45 (95.74)	>.999
Mean (SD)	374.51 (285.33)	345.97 (260.64)	405.76 (302.53)	422.83 (293.86)	354.97 (253.55)	328.84 (301.31)	
Median (IQR)	329.5 (152 – 539)	306 (109 – 506)	345 (205 – 600)	391.5 (171 – 558)	296 (122 – 573)	262 (70 – 444)	
Min, Max	6, 1496	7, 1065	8, 1496	30, 1248	6, 855	7, 1199	
Not tested (*n*)	24	6	5	5	6	2	
Psychiatric comorbidities, *n* (%)							
Yes	110 (44.72)	24 (53.33)	36 (61.02)	22 (43.14)	14 (31.82)	14 (29.79)	.009
No	136 (55.28)	21 (46.67)	23 (38.98)	29 (56.86)	30 (68.18)	33 (70.21)	
Hepatitis C, *n* (%)							
Yes	55 (22.36)	8 (17.78)	25 (42.37)	11 (21.57)	8 (18.18)	3 (6.38)	.099
No	191 (77.64)	37 (82.22)	34 (57.63)	40 (78.43)	36 (81.82)	44 (93.62)	
Syphilis, *n* (%)							
Yes	45 (18.29)	12 (26.67)	7 (11.86)	10 (19.61)	10 (22.73)	6 (12.77)	>.999
No	201 (81.71)	33 (73.33)	52 (88.14)	41 (80.39)	34 (77.27)	41 (87.23)	
Tuberculosis, *n* (%)							
Yes	27 (10.98)	5 (11.11)	5 (8.47)	6 (11.76)	4 (9.09)	7 (14.89)	>.999
No	219 (89.02)	40 (88.89)	54 (91.53)	45 (88.24)	40 (90.91)	40 (85.11)	
Pregnant at time of diagnosis, *n* (%)							
Yes	10 (4.07)	1 (2.22)	2 (3.39)	2 (3.92)	2 (4.55)	3 (6.38)	>.999
No	236 (95.93)	44 (97.78)	57 (96.61)	49 (96.08)	42 (95.45)	44 (93.62)	

Fisher exact test performed for gender, race, housing status, country region, and transmission mode. Cochran-Armitage trend test performed for ethnicity, language, diagnosing department, psychiatric comorbidities, hepatitis C, syphilis, tuberculosis, and pregnancy at time of diagnosis. *P*-values were Bonferroni-adjusted for multiple comparisons of 15 outcomes.

† Patients with no CD4 count test were excluded from summary statistics (missing CD4 count =24)

*ED*, emergency department; *IDU*, injection drug use; *IQR*, interquartile range; *MSM*, men who have sex with men; *SD*, standard deviation.

**Table 2:** Clinical characteristics of newly diagnosed patients at an urban essential hospital by primary risk factor.

Clinical Characteristics	Total Sample *N* = 246	Heterosexual Contact *n* = 146	MSM *n* = 47	IDU *n* = 46	MSM/IDU *n* = 7	*P* value
† CD4 count at time of diagnosis (cells/μL)						
Patients with available CD4 count (*n*)	222 (90.24)	131 (89.73)	42 (89.36)	42 (91.30)	7 (100)	< .001
Mean (SD)	374.51 (285.33)	285.91 (232.23)	376.19 (234.70)	603.55 (339.58)	648.29 (199.19)	
Median (IQR)	329.5 (152 – 539)	245 (91 – 416)	396.5 (226 – 502)	579 (304 – 855)	613 (574 – 639)	
Min, Max	6, 1496	6, 1199	7, 1053	45, 1496	413, 1065	
Not tested (*n*)	24	15	5	4	0	
Psychiatric comorbidities *n* (%)						< .001
Yes	110 (44.72)	35 (23.97)	23 (48.94)	45 (97.83)	7 (100)	
No	136 (55.28)	111 (76.03)	24 (54.06)	1 (2.17)	0 (0.0)	
Hepatitis C *n* (%)						< .001
Yes	55 (22.36)	3 (2.05)	2 (4.26)	45 (97.83)	5 (71.43)	
No	191 (77.64)	143 (97.95)	45 (95.74)	1 (2.17)	2 (28.57)	
Syphilis *n* (%)						< .001
Yes	45 (18.29)	17 (11.64)	24 (51.06)	3 (6.52)	1 (14.29)	
No	201 (81.71)	129 (88.36)	23 (48.94)	43 (93.48)	6 (85.71)	
Tuberculosis *n* (%)						> .99
Yes	27 (10.98)	19 (13.01)	3 (6.38)	5 (10.87)	0 (0.0)	
No	219 (89.02)	127 (86.99)	44 (93.62)	41 (89.13)	7 (100)	

*P* values were Bonferroni-adjusted for multiple comparisons of five outcomes. Chi-square (categorical) and one way ANOVA (continuous) were performed.

† Patients with no CD4 count test were excluded from summary statistics (missing CD4 count, 24)

*IDU*, injection drug use; *IQR*, interquartile range; *MSM*, men who have sex with men; *MSM/IDU*, men who have sex with men and injection drug use; *SD*, standard deviation.

**Table 3:** Pairwise comparisons of the secondary outcome CD4 cell count at time of diagnosis by demographic characteristics in a study assessing changes in transmission risk factors among patients newly diagnosed with HIV.

Pairwise Comparisons of Demographic Characteristics	Difference between mean CD4 cell count (cells/μL)	Adjusted 95% confidence interval for difference between two mean CD4 cell counts (cells/μL)	*P* value*
Country of origin			
Non-US-born – US-born Patients	198.8	(125.4, 272.2)	.001
Diagnosing department			
Non-ED patient – ED patient	144.5	(59.0, 230.0)	.005
Housing status			
Not stable – Stable	164.0	(61.3, 266.8)	.003
Not stable – Unknown	200.8	(−51.3, 452.8)	> .99
Stable – Unknown	36.7	(−205.5, 279.0)	> .99

* *P* values were Bonferroni-adjusted for multiple pairwise comparisons (five comparisons).

*ED*, emergency department.
