# Supplementary material for: Changing Risk Factors in Patients Diagnosed with Human Immunodeficiency Virus
Source: West J Emerg Med. 2026 Aug 26;27(5):1402–10. doi: 10.5811/westjem.52947 (PMC13660644; doi:10.5811/westjem.52947)
Supplement: Supplementary file 1 [file wjem-27-1402-s001.docx]

**Supplemental Table 2**: **Pairwise comparisons of CD4 count at time of diagnosis for Primary HIV Risk Factors**

| **Pairwise Comparisons of HIV Risk Factors** | **Mean Difference (cells/µL)** | **Adjusted 95% CI for difference between two means (cells/µL)** | ***P-*value*** |
| --- | --- | --- | --- |
| Heterosexual - IDU | -317.6 | (-438.3, -197.0) | < .001 |
| Heterosexual - MSM | -90.3 | (-210.9, 30.4) | > .99 |
| Heterosexual - MSM/IDU | -362.4 | (-626.3, -98.4) | .01 |
| IDU - MSM | 227.4 | (78.9, 375.8) | .002 |
| IDU - MSM/IDU | -44.7 | (-322.5, 233.0) | > .99 |
| MSM - MSM/IDU | -272.1 | (-549.9, 5.7) | .35 |

*** *P*-values and 95% CI were Bonferroni adjusted for multiple pairwise comparisons (6 comparisons).**

**HIV, human immunodeficiency virus; MSM, men who have sex with men; IDU injection drug use; MSM/IDU, men who have sex with men and injection drug use**
