# Supplementary material for: Changing Risk Factors in Patients Diagnosed with Human Immunodeficiency Virus
Source: West J Emerg Med. 2026 Aug 26;27(5):1402–10. doi: 10.5811/westjem.52947 (PMC13660644; doi:10.5811/westjem.52947)
Supplement: Supplementary file 2 [file wjem-27-1402-s002.docx]

**Supplemental Table 3: CD4 cell counts of newly diagnosed patients at an urban essential hospital by Country of Origin, Diagnosing Department, and Housing Status**

|  | **† CD4 count at time of diagnosis (cells/µL)** | | | | | |
| --- | --- | --- | --- | --- | --- | --- |
| **Demographic Characteristics (n=246)** | **Total for CD4 count (n=222)** | **Mean (SD)** | **Median (IQR)** | **Min, Max** | **Not tested (n)** | **P-value** |
| **Country of Origin** |  |  |  |  |  | < .001 |
| U.S.-born Patients (n=94) | 84 | 498.08 (309.45) | 466.5 (270 - 676.5) | 7, 1496 | 10 |  |
| Foreign Patients (n=152) | 138 | 299.29 (241.35) | 250 (93 - 444) | 6, 1199 | 14 |  |
| **Diagnosing Department** |  |  |  |  |  | .003 |
| ED patient (n=63) | 55 | 265.78 (262.67) | 210 (38 - 413) | 6, 1065 | 8 |  |
| Non-ED patient (n=183) | 167 | 410.32 (284.14) | 377 (205 - 580) | 8, 1496 | 16 |  |
| **Housing Status** |  |  |  |  |  | .002 |
| Stable (n=171) | 156 | 322.97 (244.79) | 318.5 (124.5 - 492.5) | 7, 1199 | 15 |  |
| Unstable (n=63) | 58 | 497.02 (334.96) | 488.5 (245 - 678) | 6, 1496 | 5 |  |
| Unknown (n=12) | 8 | 296.25 (400.03) | 163.5 (83.5 - 305.5) | 17, 1248 | 4 |  |

**† Patients with no CD4 count test were excluded from summary statistics (missing CD4 count = 24)**

**P-values were Bonferroni-adjusted for multiple comparisons of 3 outcomes**

**U.S., United States; ED, emergency department**
